# Spike Spectra for Recurrences

**DOI:** 10.3390/e24111689

**Published:** 2022-11-18

**Authors:** K. Hauke Kraemer, Frank Hellmann, Mehrnaz Anvari, Jürgen Kurths, Norbert Marwan

**Affiliations:** 1 Potsdam Institute for Climate Impact Research (PIK), Member of the Leibniz Association, 14473 Potsdam, Germany; 2 Institute of Physics and Astronomy, University of Potsdam, 14476 Potsdam, Germany; 3 Institute of Physics, Humboldt Universität zu Berlin, 12489 Berlin, Germany; 4 Institute of Geosciences, University of Potsdam, 14476 Potsdam, Germany

**Keywords:** decomposition, frequency analysis, recurrence analysis, bifurcations, 05.45.Tp, 05.90.+m, 89.90.+n, 02.70.Uu, 05.10.Ln, 05.45.-a, 05.45.Ac

## Abstract

In recurrence analysis, the τ-recurrence rate encodes the periods of the cycles of the underlying high-dimensional time series. It, thus, plays a similar role to the autocorrelation for scalar time-series in encoding temporal correlations. However, its Fourier decomposition does not have a clean interpretation. Thus, there is no satisfactory analogue to the power spectrum in recurrence analysis. We introduce a novel method to decompose the τ-recurrence rate using an over-complete basis of Dirac combs together with sparsity regularization. We show that this decomposition, the *inter-spike spectrum*, naturally provides an analogue to the power spectrum for recurrence analysis in the sense that it reveals the dominant periodicities of the underlying time series. We show that the inter-spike spectrum correctly identifies patterns and transitions in the underlying system in a wide variety of examples and is robust to measurement noise.

## 1. Introduction

The dynamics of complex systems as provided by measured time series usually show complicated and chaotic patterns. Quantifying their recurrence features is a powerful way to describe them and to infer information about the type of dynamics, stability, regime changes, or couplings and synchronisation [1,2,3]. Even more challenging are signals which do have a heavy tailed-distribution or appear as a spike-train, e.g., neuron firings [4,5], heart beat variability [6], or extreme flood events [7]. Deriving useful information from spike-train signals or inter-spike time series is an important topic in data analysis in many scientific fields [4,5,8,9].

Recurrence plots (RPs) provide a vivid representation of complex dynamics ${\vec{x}}_{i}$ stemming from potentially high dimensional systems [1]

(1) $$R_{i,j}\left(\varepsilon \right)=\Theta \left(\varepsilon -D_{i,j}\right)=\Theta \left(\varepsilon -∥{\vec{x}}_{i}-{\vec{x}}_{j}∥\right),\;\vec{x}\in R^{d},\;i,j\in \left[1,\ldots ,N\right],$$

where R is the recurrence matrix, ${\vec{x}}_{i}$ is the state vector at time t=Δt·i (Δt the sampling time), *N* is the number of sampling points (or length of data series), and *d* is the dimension of the system. The crucial free parameter ε is the recurrence threshold, determining what is a recurrence and, thus, the visible structures in the RP. It can be chosen such that the recurrence rate $RR\left(\varepsilon \right)=N^{-2}\sum_{i,j}^{N}R_{i,j}\left(\varepsilon \right)$ exhibits a certain value [10]. The simple idea to track recurring states of the *d*-dimensional trajectory ${\vec{x}}_{i}$ of the system under study not only allows for a beneficial visualization of the dynamics, but also for its quantification, using certain structures in the RP, such as diagonal or vertical lines [1].

Some of these recurrence quantification measures, the entropy of diagonal lines and the entropy of recurrence times, can be related to the basic characteristics of complex systems, such as Kolmogorov–Sinai entropy [11,12]. However, these quantifiers have a free parameter, the minimal considered line length, and are usually biased, due to the finite size of the RP and thickened diagonal lines, which need to be corrected [13]. Moreover, the mentioned statistics cannot account for changing regular (non-chaotic) dynamics, such as period-doubling bifurcations.

A rather simple idea is to look at the τ-recurrence rate of the RP (τ-RR, Equation (2)) [14,15]. This is the density of recurrence points along the diagonals of the recurrence matrix, as a function of the distance τ (sampling units) to the main diagonal:

(2) $$\tau -\mathrm{RR}\left(\varepsilon \right)=RR\left(\tau ,\varepsilon \right)=\frac{1}{N-\tau }\sum_{i=1}^{N-\tau }R_{i,i+\tau }.$$

**Figure 1:**
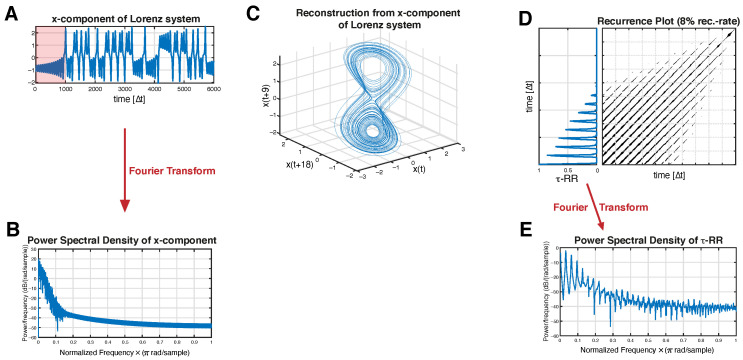
Schematic illustration of a τ-recurrence-rate-based spectrum. (**A**) *x*-component time series of the Lorenz63-System (Equation (A1)) and (**B**) its corresponding Fourier power spectrum. (**C**) Reconstructed state space portrait from the time series shown in (**A**) using PECUZAL time-delay embedding [16]. (**D**) Subset of the recurrence plot and the corresponding τ-recurrence rate obtained from the state space trajectory in (**C**). (**E**) Fourier Power spectrum obtained from the τ-recurrence rate (subset shown in panel (**D**)) [15]. (**D**,**E**) show the results of a part of the time series, which is highlighted in pink in (**A**).

τ-RR serves as an estimator for the probability that the system recurs after time τΔt, with Δt being the sampling time of the trajectory ${\vec{x}}_{i}=\vec{x}\left(\Delta t\;\cdot \;i\right),\;i=1,\ldots ,N$. It represents the period length of cycles in the data (Figure 1D).

Zbilut and Marwan [15] found that τ-RR could be interpreted as analogous to the auto-correlation function C(τ) and, hence, via the Wiener–Khinchin theorem, provide an analogue, “generalized”, spectral density. This is reasonable, since the average distances for a given lag τ

(3) $$\bar{D}\left(\tau \right)=\frac{1}{N-\tau }\sum_{i=1}^{N-\tau }D_{i,i+\tau }$$

can be directly read from the distance matrix D and are also preserved in its thresholded version τ-RR. There are clear advantages to recurrence-derived spectral density, i.e., Fourier transforming (FT) τ-RR (Figure 1D,E), instead of C(τ). There are no assumptions for stationarity or sampling when constructing a RP. Furthermore, since an RP can represent high-dimensional dynamics and its τ-RR serves as a plug-in for C(τ), the correlation structures of higher dimensional spaces can be read from the recurrence-derived Fourier spectrum.

However, the interpretation of this generalized power spectral density is unclear, and it is typically hard to interpret. τ-RR directly encodes the periodicity of the underlying signal; in contrast, it is unclear what, if any, interpretation the “power” contained in a particular frequency mode of the τ-RR should be. Whenever τ-RR is a spike-train-like signal, which it is in most cases (see Figure 1) especially for map-data (low-resolution data), an FT of such a signal leads to a spike-train-like image in the frequency domain (e.g., [17,18], see Figure 1E). Thus, how to extract meaningful information about the systems’ state space trajectory is not intuitive.

**Figure 2:**
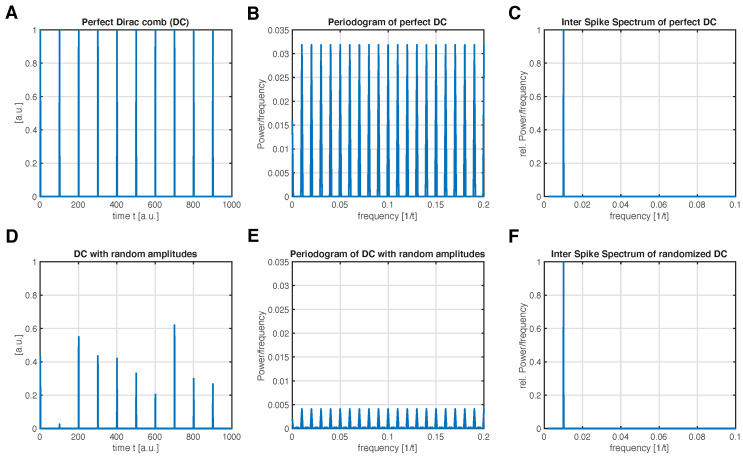
The transformation of a Dirac comb (series of Dirac delta functions) with a single inter-spike period $T_{\mathrm{is}}=100$ ($\hat{=}f=0.01$) into the frequency domain. (**A**) Dirac Comb (DC) with equal amplitudes and (**B**) its FFT-based power spectral density. (**C**) Proposed inter-spike spectrum of the signal in (**A**) showing a single frequency, which corresponds to the inter-spike period $T_{\mathrm{is}}$ (f=0.01). (**D**) DC with randomly chosen amplitudes and same $T_{\mathrm{is}}$ as in (**A**), and (**E**) is its FFT-based power spectral density. (**F**) Proposed inter-spike spectrum of the signal in (**D**) showing a single frequency, which corresponds to the expected inter-spike period $T_{\mathrm{is}}$ (f=0.01). Inter-spike spectra were obtained with a LASSO regression and a regularization threshold corresponding to ρ=0.9 accordance of the signals in (**A**,**D**) and its re-composed signals (c.f. Section 2).

For example, consider the signal we would like to analyze (e.g., the τ-RR of a system) to be a Dirac comb (DC) with inter-spike period $T_{\mathrm{is}}$:

(4) $${\mathrm{DC}}_{\mathrm{is}}\left(t\right)=\sum_{k=-\infty }^{\infty }\delta \left(t-kT_{\mathrm{is}}\right),$$

i.e., a series of Dirac delta functions for a period $T_{\mathrm{is}}$. There is only one single period, $T_{\mathrm{is}}$, in this signal (Figure 2A,D); in principle, we would strive for a single peak in the frequency domain of this signal at frequency $f=1/T_{\mathrm{is}}$. The Fourier spectrum does not meet this expectation and instead of a single frequency, there are many excited frequencies (Figure 2B,E). This is because the Fourier components constructively contribute to every frequency $1/T_{\mathrm{is}}$; therefore, ${\mathrm{DC}}_{\mathrm{is}}\left(t\right)$ coincides with its own Fourier transform up to a factor $1/T_{\mathrm{is}}$ [19]. (This can also be observed for neuron spike trains, e.g., [5,20].)

In this article, we propose a new way of decomposing a spike-train-like signal into periodic components as an alternative to the RP-based method suggested in [15] or Fourier-based spike-train power spectra [4]. This novel *inter-spike spectrum* does not show resonance behavior of the signal’s inherent inter-spike frequencies in such a way that the harmonics of these frequencies are also excited (Figure 2C,F). Section 2 explains the idea. Note that this approach can be used to decompose arbitrary signals, and is not specific to τ-RR. However, the more spiky the signal is, the more useful our new approach is compared to FT. In Section 3, we demonstrate the usage of inter-spike spectrum when transforming the τ-RR of a system under study. In this case, the inter-spike spectrum can unravel characteristic time scales of high dimensional systems, which is not possible when using an FT. Finally, in Section 5 our results are summarized.

## 2. Method

To obtain the inter-spike spectrum, the signal, in our case the τ-RR, is decomposed into a set of appropriate basis functions. The general idea common to many methods is that the sum of these weighted functions can approximate a finite signal to a sufficient degree. The weights (in some contexts also called modes or loadings) corresponding to the individual basis functions must be determined. A number of decomposition techniques based on different sets of basis functions exist, e.g., trigonometric functions (Fourier and wavelet analysis [21]), eigenvectors of the corresponding covariance matrix (principal component analysis [22] and related techniques) or intrinsic mode functions (empirical mode decomposition and Hilbert spectrum [19]). These methods typically share the property that the basis functions form a complete basis, and the set of basis functions is linearly independent; thus, the weights are uniquely determined.

**Figure 3:**
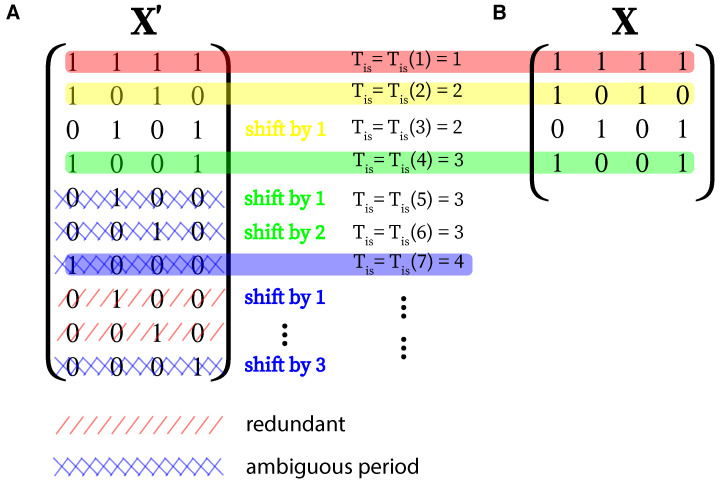
(**A**) Example of a full set of basis functions for an input signal of length N=4, aligned in the matrix $X^{\prime }$. Inter-spike periods $T_{\mathrm{is}}$ larger than ⌈N/2⌉+1 lead to redundant basis functions (i.e., repeated lines in **X**, red sheared) or basis functions, which cannot be uniquely assigned to a certain inter-spike period (blue sheared). $T_{\mathrm{is}}$ for each row can be obtained by Equation (6). (**B**) The final set of unambiguous, but still linearly dependent, basis functions aligned in the matrix **X**.

Here, we propose the use of Dirac combs (DC) with different inter-spike periods as basis functions, as shown in Equation (4). Let $s(t_{i})$ be the min-max-normalized signal we want to transform in terms of length *N* and $t_{i}=i\;\cdot \;\Delta t,\;i=1,\ldots ,N$, where Δt denotes the sampling time and $s\left(t_{i}\right)\in \left[0,\;1\right]\;\forall \;i$. In the following sections, we label this time series as a (1×N)-dimensional vector $\vec{s}$. First, $\tilde{N}$ different DCs of length *N* are constructed with inter-spike periods $T_{\mathrm{is}}\in \left[1,\ldots ,\tilde{N}\right]$ and $\tilde{N}=N/2+1$. Second, in order to account for possible phase shifts of these basis functions occurring in $\vec{s}$, each of these $\tilde{N}$ different DCs also need to be shifted one step further $T_{\mathrm{is}}-1$ times. This leaves us with a total number of $M=\sum_{i=1}^{\tilde{N}}i$ basis functions which we can arrange as rows of a (M×N)-sized matrix (Figure 3 illustrates the described procedure) as follows:

(5) $$\begin{array}{cc} X_{i,j} & =\sum_{k=0}^{N}\delta \left(j-1-kT_{\mathrm{is}}\left(i\right)-i+T_{\mathrm{is}}\left(i\right)\right),\;i=1,\cdots ,\left\lceil N/2\right\rceil +1,\;j=1,\cdots ,N \end{array}$$

(6) $$\begin{array}{cc} T_{\mathrm{is}}\left(i\right) & =n,\;\mathrm{such}\;\mathrm{that}\;n:\;\frac{n(n-1)}{2}+1\leq i<\frac{n(n+1)}{2}+1,\;n\in N_{+}. \end{array}$$

Note that due to the shifting of each of the basis functions of the inter-spike period $T_{\mathrm{is}}X$, is no longer linear-independent. Furthermore, there will be identical basis functions, which do not allow for an unambiguous inter-spike period, if we would include all *N* possible inter-spike periods for a signal of length *N* instead of ⌈N/2+1⌉ (Figure 3A). The reason is that in contrast to a trigonometric decomposition, where the Nyquist frequency marks a lower bound for the corresponding wave period, the maximum considered inter-spike period is bounded by $T_{\mathrm{is}}^{\max}=\left\lceil N/2\right\rceil +1$ (schematically illustrated in Figure 3B).

Eventually, an under-determined linear system

(7) $$X^{T}\beta =\vec{s}$$

has to be solved for β, with the (M×1)-sized vector carrying the loadings we are interested in. Of the variety of algorithms which can solve this problem, we are particularly interested in those solutions which promote sparsity in β, since our goal is to decompose the signal $\vec{s}$ into a minimal number of basis functions (for an excellent overview of the topic we refer readers to Brunton and Kutz [23]). In this paper, we either use the *least absolute shrinkage and selection operator* (LASSO) [24] or a *sequentially threshold least squares* (STLS) regression [23,25] to obtain a solution $\hat{\beta }$. Any other sparse regression technique can be used. Finally, we group loadings which correspond to basis functions with the same period $T_{\mathrm{is}}$ into ${\hat{\beta }}_{f}$ and obtain the *inter-spike spectrum* by simply plotting ${\hat{\beta }}_{f}$ as a function of the frequency $f=T_{\mathrm{is}}^{-1}$, with $T_{\mathrm{is}}=\Delta t,2\Delta t,\cdots ,\left(\left\lceil N/2\right\rceil +1\right)\Delta t$ (Figure 2C,F).

When introducing a sparsity regularization, we forsake the ability to fully reconstruct the signal from the decomposition in favor of identifying the most prominent spikes, and thus, the most important periodicities, more accurately. This choice is parametrized by a regression regularization parameter α. We only obtain a solution to Equation (7) with α=0 as well as a full reconstruction of the signal. Our general approach to fixing α is to preserve most of the original signal in the sense of matching a given (Pearson) correlation coefficient ρ between the original signal $\vec{s}$ and the reconstruction. The chosen regression method (in our case, LASSO or STLS) will determine how sensitive the obtained inter-spike spectrum is with respect to the regularization of the selected ρ (Figure A5). Regarding this, in most of our applications LASSO yields more robust results, while STLS is usually faster in terms of computation. We will discuss this in more detail in Section 4. The proposed decomposition does not show a leakage effect by construction. However, when introducing sparsity regularization we forsake the ability to fully reconstruct the signal from the decomposition in favor of identifying the most important periodicities more clearly. This choice is mediated by a regression regularization parameter α. Only for α=0 do we obtain a solution to Equation (7) and thus a full reconstruction of the signal. Our general approach to fixing α is to preserve most of the original signal in the sense of matching a given (Pearson) correlation coefficient ρ between the original signal $\vec{s}$ and the reconstruction. The chosen regression method (in our case, LASSO or STLS) will determine how sensitive the obtained inter-spike spectrum is with respect to the regularization achieved by a selected ρ (Figure A5). Regarding this, in most of our applications LASSO yields more robust results, while STLS is usually faster in terms of computation. We will discuss this in more detail in Section 4.

## 3. Results

We demonstrate the use of the inter-spike spectrum in combination with τ-RR, as outlined in Section 1, on several interesting research questions. The procedure takes place as follows:

(1) Compute an RP of the trajectory of the system, Equation (1). If only univariate data are available, perform a state space reconstruction for obtaining the trajectory first.
(2) Compute the τ-RR of the RP, as shown in Equation (2).
(3) Transform the τ-RR into the proposed inter-spike spectrum, see Section 2.

### 3.1. Period Estimation for Different Dynamics in the Rössler System

**Figure 4:**
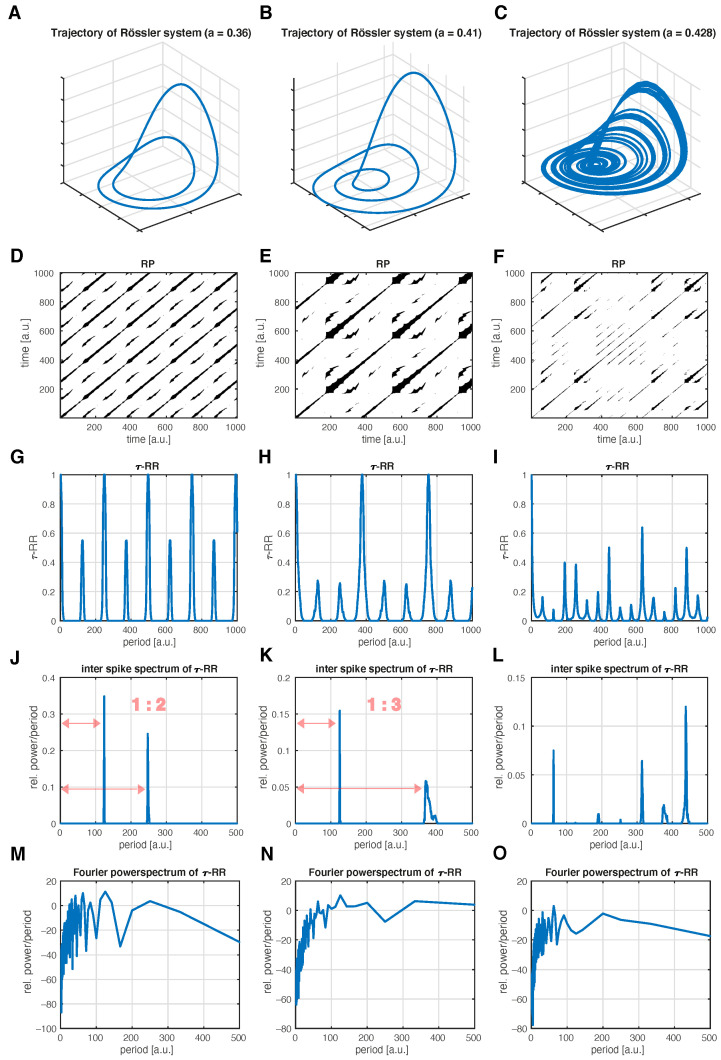
Inter-spike spectra of the τ-RR of the Rössler system in three different dynamical regimes with parameters b=2, c=4. (**A**) Trajectory of the system in a period-2 (parameter a=0.36), (**B**) in a period-3 (parameter a=0.41) and (**C**) in a chaotic regime (parameter a=0.428). (**D**–**F**) The corresponding RPs, obtained by using a recurrence threshold corresponding to a 10% global recurrence rate for (**D**,**E**) and 5% for (**F**). (**G**–**I**) τ-RRs of the shown RPs. (**J**–**L**) The proposed inter-spike spectra of the τ-RRs shown in panels (**G**–**I**). Spectra were obtained with a LASSO regression and a regularization threshold corresponding to ρ=0.95 accordance of τ-RRs and re-composed signals. The distance ratio of the peaks reflect the limit cycle dynamic. (**M**–**O**) Fourier power spectra of the τ-RRs shown in panels (**G**–**I**).

First, we consider the Rössler system (Equation (A2), shown in Appendix A.2), in three different dynamical setups. We use the proposed inter-spike spectrum to identify the type of dynamics. We set the parameters as b=2, c=4 and analyze period-2 limit cycle dynamics (a=0.36 in Figure 4A,D,G,J), period-3 limit cycle dynamics (a=0.41 in Figure 4B,E,H,K) and chaotic dynamics (a=0.428 in Figure 4C,F,I,L).

**Figure 5:**
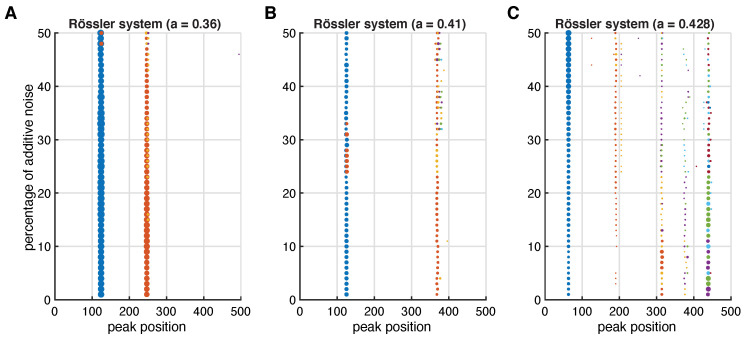
Peak positions of the obtained inter-spike spectra of the τ-RRs for additive noise levels up to 50% for the discussed Rössler dynamics, (**A**) period-2 limit-cycle, (**B**) period-3 limit-cycle and (**C**) chaos. The size of the plotted markers scale with the detected peak height. The noise-free spectra are shown in Figure 4J–L and an example of these spectra with 5% additive noise is shown in Figure A3J–L. Spectra were obtained with a LASSO regression and a regularization threshold corresponding to ρ=0.95 accordance of τ-RRs and re-composed signals.

The inter-spike spectra unravel the specific dynamics, which are also apparent in the state space portraits (Figure 4A–C) and in the τ-RRs (Figure 4G–I). The proposed idea is also robust to noise (see Figure A3 in the Appendix C). This is because the peaks of τ-RR are insensitive to noise. Figure 5 illustrates that while the peak shape does change in the presence of noise, its position does not, and this is what the inter-spike spectrum encrypts (see Figure A4 in the Appendix C for further analysis).

### 3.2. Bifurcations in the Logistic Map

We consider the Logistic map $x_{n+1}=r\;\cdot \;x_{n}\left(1-x_{n}\right)$ for changing the control parameter *r*. We vary *r* from r=3.4 to r=4 in steps of 0.001. For each setting of *r*

(1) A time series of length N=201 is computed with a random initial condition $u_{0}\in \left[0,\;1\right]$, neglecting the first 1000 samples as transients;
(2) A total of 100 iterative Amplitude Adjusted Fourier Transform (iAAFT) surrogates [26,27] are computed;
(3) The time series and its iAAFT surrogates are embedded in a 2-dimensional state space using a time delay of unity;
(4) The two-dimensional trajectories RPs, as shown in Equation (1), are computed under a threshold ε=0.05,
(5) τ-RR, as shown in Equation (2), is computed from the RP of the signal and from the RPs of the surrogates;
(6) Inter-spike spectra are obtained from τ-RR of the signal and from the τ-RRs of the surrogates, as can be seen in Section 2;
(7) Finally, from the distribution of the surrogate inter-spike spectra, the 95th percentile is computed. The peaks of the inter-spike spectrum of the signal which exceed this percentile are counted.

**Figure 6:**
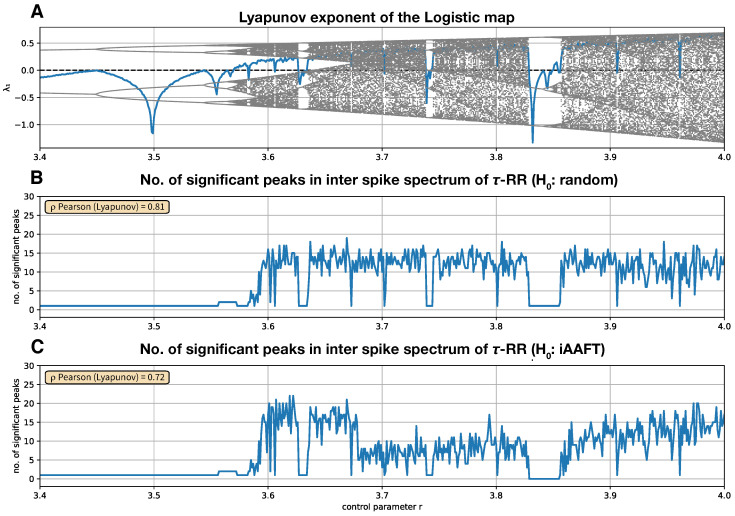
(**A**) Bifurcation diagram and Lyapunov exponent and of the Logistic map as a function of the control parameter *r*. (**B**) Number of significant peaks (α=0.05) in the inter-spike spectrum of the τ-RR and its Pearson correlation coefficient to the Lyapunov exponent shown in (**A**) (white noise surrogates). (**C**) Same as (**B**), but for iterative Amplitude Adjusted Fourier Transform (iAAFT) surrogates [26,27]. To obtain the inter-spike spectra, we used a LASSO regression and a regularization threshold corresponding to the ρ=0.95 accordance of τ-RRs and re-composed signals.

In this example, the null hypothesis for constructing the surrogate data is that the data stem from a process which yields the same auto-correlation, and hence, the same Fourier power spectral density, and the same amplitude distribution. We consider the number of significant peaks in the inter-spike spectrum with respect to the control parameter in order to distinguish the corresponding dynamics (Figure 6C). A correlation with the positive Lyapunov exponent (Figure 6A) is discernible ($\rho_{\mathrm{Pearson}}\left(\mathrm{Lyapunov}\right)=0.72$). This analysis can handle period-doubling since it measures the dominant cycles via the inter-spike spectrum. However, whenever the periods of the new cycles coincide with integer multiples of the periods of already existing cycles, the approach cannot detect period doubling. A similar case can be observed in Figure 4J,K, where the number of peaks does not change, but the mutual distance does.

A less computationally intensive approach is to compute surrogates for τ-RR analytically, rather than computing an RP and its τ-RR for each iAAFT surrogate of the time series. This translates into a null hypothesis that the τ-RR and its corresponding inter-spike spectrum stem from an RP of a random signal. In this case, the probability of finding a black point in the RP can be obtained from a binomial distribution with the probability parameter *p* set to the recurrence rate of the RP of the signal. In this way, 100 surrogate τ-RRs are computed in step (5). The results are even slightly better compared to the ones obtained from the iAAFT surrogates (Figure 6B, $\rho_{\mathrm{Pearson}}\left(\mathrm{Lyapunov}\right)=0.81$). The first instance of period doubling at r≈3.458 cannot be detected by any of the surrogates.

The described procedure does work well for map data, because most often the τ-RR for those kind of data reveals a “spiky enough” nature. On the contrary, highly sampled (flow-) data often yield not as spiky τ-RRs; therefore, the number of significant peaks in the inter-spike spectrum may not be sensitive enough to detect period-doubling bifurcations. Moreover the sensitivity of the inter-spike spectrum in detecting regime shifts also depends on the critical regularization threshold. Nevertheless, the according inter-spike spectra still reveal important information (Figure 4) and practitioners can design appropriate quantifying statistics based on these spectra which suit the research task.

### 3.3. Inter-Spike Spectra of Power Grid Frequency Data

Power grids are large, synchronized, complex networks whose stable functioning is indispensable for modern societies. To maintain the stability of a power grid, the balance between energy consumption and energy generation must be ensured. In an AC-power grid, the grid frequency is an observable variable that reflects how well this balance is satisfied. In this process, the grid frequency and its deviations from the nominal frequency are continuously recorded and monitored by the grid operators (in Europe and many parts of the world this is 50 Hz or 60 Hz in America and, for example, southern Japan). For example, if there is more (less) demand than supply, the network frequency decreases (increases) compared to the nominal frequency [28].

**Figure A1:**
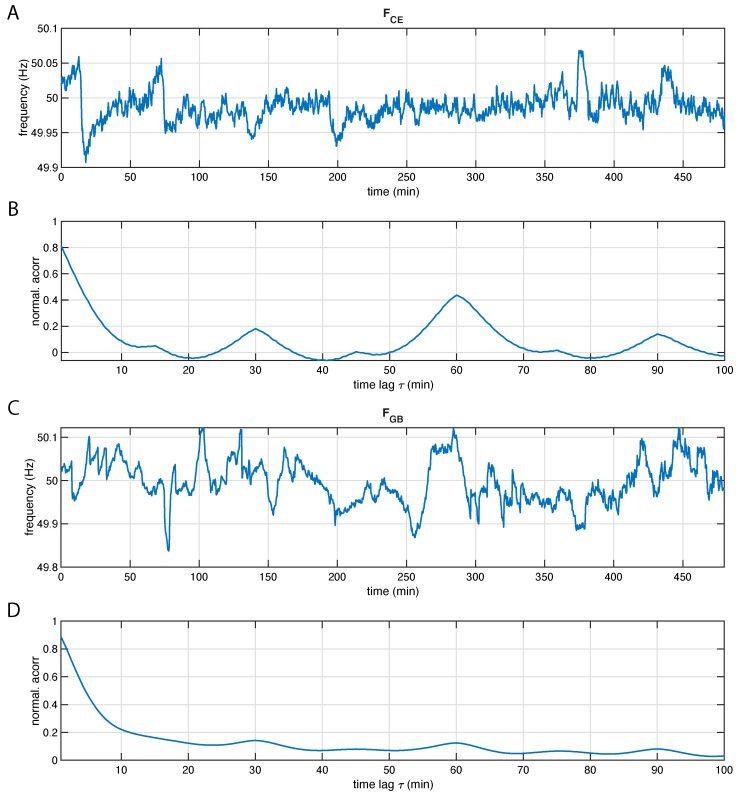
Subset of the frequency time series of (**A**) Central Europe (CE) [45] and (**C**) Great Britain (GB) [44] along with their autocorrelation functions in (**B**,**D**), respectively. The normalized autocorrelations in (**B**,**D**) were computed with the entire time series, whereas the subsets shown in (**A**,**C**) contain only 1441 samples.

**Figure A3:**
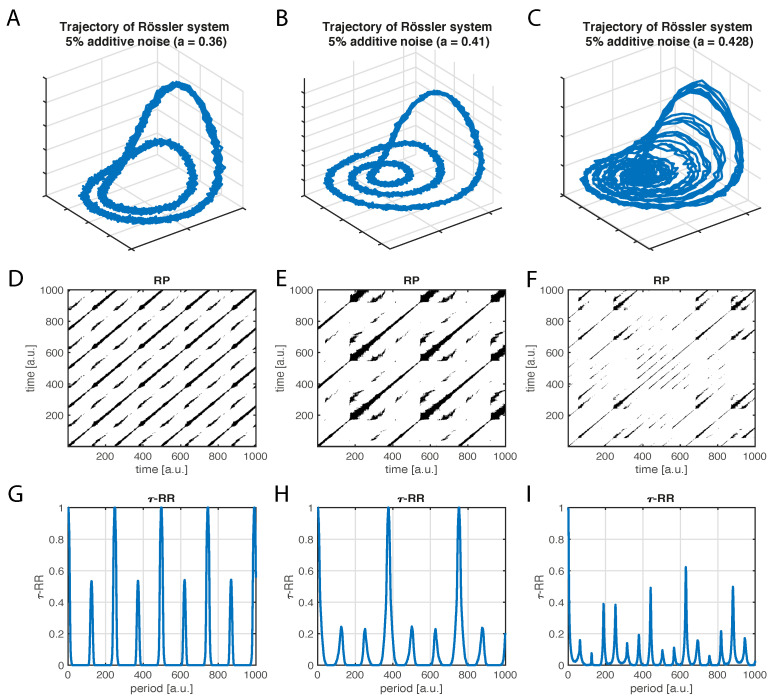

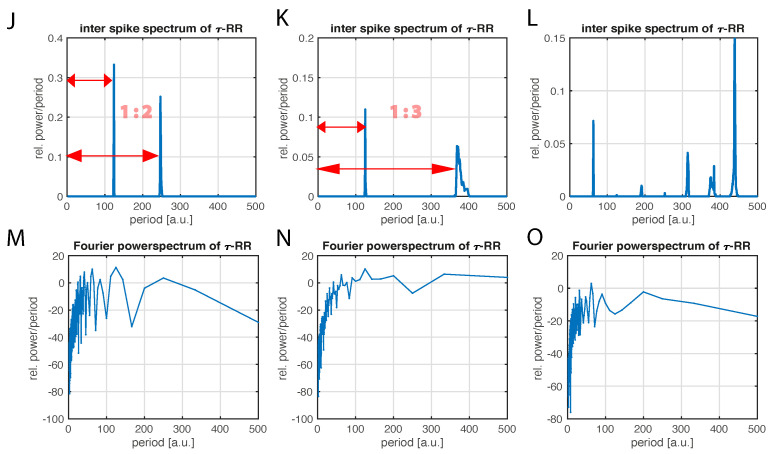
Same as in Figure 4, but here with 5% additive Gaussian white noise on each component *x*, *y* and *z*. (**A**) Trajectory of the system in a period-2 (parameter a=0.36), (**B**) in a period-3 (parameter a=0.41) and (**C**) in a chaotic regime (parameter a=0.428). (**D**–**F**) The corresponding RPs, obtained by using a recurrence threshold corresponding to a 10% global recurrence rate for (**D**,**E**) and 5% for (**F**). (**G**–**I**) τ-RRs of the shown RPs, (**J**–**L**) the corresponding inter-spike spectra, and (**M**–**O**) the Fourier power spectra. The appearance of the inter-spike spectra in (**J**–**L**), and the Fourier spectra in (**M**–**O**) are unaffected by the additive noise.

**Figure A4:**
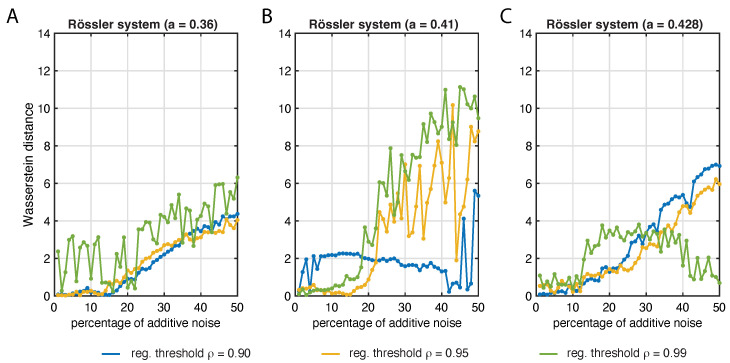
Wasserstein distances for the obtained inter-spike spectra of the τ-RRs for additive noise levels of up to 50% for the discussed Rössler dynamics, (**A**) period-2 limit-cycle, (**B**) period-3 limit-cycle and (**C**) chaos. For each noise level the obtained spectrum is compared to the noise-free spectrum (these are shown in Figure 4J–L for a regularization threshold corresponding to ρ=0.95) by computing its Wasserstein distance. Inter-spike spectra were obtained with a LASSO regression and three different regularization thresholds corresponding to ρ=0.9 (blue), ρ=0.95 (orange) and ρ=0.99 (green) accordance of τ-RRs and re-composed signals. Up to a noise level of 20% the distances are small and rather constant. The fluctuations depend on the chosen regularization threshold as well as on the underlying dynamics.

The frequency variations can include other information, such as the functionality of control systems [29], the effect of fluctuations in renewable energies (REs), demands on the grid [30] and, moreover, the effect of regular dispatches due to the trading market [31]. The latter induce periodic frequency jumps. Here we look at the frequency time series for the Great Britain (GB) and Continental Europe (CE) (Appendix B and Figure A1A,C). Clear jumps every 30 and 60 min are discernible and quantitatively reflected in the corresponding autocorrelations (Figure A1B,D). Furthermore, the autocorrelation of the CE frequency time series shows regular peaks every 15 min (see Figure A1B). These peaks are caused by a mismatch of power supply and demand [32] during dispatches. In most electricity grids the operation of dispatchable power plants is scheduled in 1-h blocks, where additional (shorter) 30 and 15 min intervals might exist.

**Figure 7:**
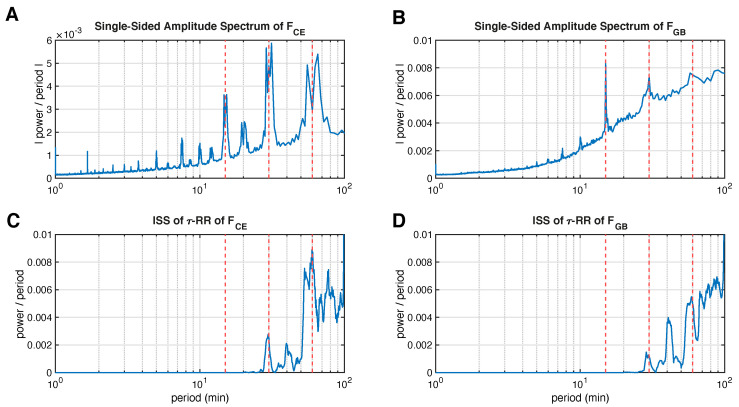
Averaged evolutionary Fourier power spectra of recorded power grid frequency time series of (**A**) Central Europe (CE) and (**B**) Great Britain (GB) (see Figure A1 in Appendix B). The corresponding averaged inter-spike spectra of the according τ-RRs, Equation (2), are shown in (**C**,**D**), respectively. Vertical red dashed lines correspond to 15, 30 and 60 min. For technical details on the calculation of the spectra shown, i.e, the preprocessing and window size being used, the reader is referred to Appendix B.

The evolutionary Fourier frequency spectrum for the Central European data in Figure 7A, however, does not display sharp peaks exactly at 15, 30 and 60 min, which may partly be explained by the leakage effect (for technical details on the calculation of the spectra shown, the reader is referred to Appendix B). More clearly, the 30 and 60 min peaks are split into two adjacent peaks and the local minimum in between these “double”-peaks correspond to the exact times. The Great Britain counterpart in Figure 7B has sharp peaks at 15 and 30 min and also “local predecessor peaks” for 30 and 60 min at the same positions as in panel A. The fact that sharp peaks are seen here using the same window size and sampling frequency as in the case of the Central European data shows that the leakage effect alone is not the cause of the peak splitting, as shown in Figure 7A.

In contrast, the 15 min peak is completely absent from the inter-spike spectra of the τ-RRs of the frequency data (Figure 7C,D), but these show sharp peaks at 30 and 60 min (there is no leakage effect due to the proposed decomposition technique). In front of these peaks, smaller local peaks can be seen, which correspond to the local peaks of the Fourier power spectra at 28 and 55 min, respectively. Moreover, there is an additional peak at 40 min for both datasets, which is absent in the Fourier spectrum and which is not a multiple of the missing 15 min peak. The position and magnitude of the peaks in the inter-spike spectra are robust to the chosen recurrence threshold, the regression method and its regularization as well as the sampling time of the original signal.

We interpret the results presented as follows. The missing 15 min in the inter-spike spectra is due to the much stronger autocorrelation at 30 and 60 min (see Figure A1B,D); because these periods are integer multiples of 15 min, the inter-spike spectra are not able to detect them and the sparse regression “drags” the 15 min periods into the mentioned 30 and 60 min peaks. Moreover, the inter-spike spectra, unlike the Fourier spectra, demonstrate sharp peaks exactly at 30 and 60 min, i.e., during dispatches (not valid in case of the GB dataset). Finally, we found a clear sharp peak at 40 min which can occur because of any regular controls in a power grid, and can indicate the need to develop the existing stochastic processes to model the power grid frequency more precisely [29].

### 3.4. Evolutionary Inter-Spike Spectra of Earth’s Orbit Data

When applying the proposed inter-spike spectrum to the τ-RR of a time series we expect additional frequency/period information, due to the fact that the recurrence plot (RP), Equation (1), and its corresponding τ-RR, as shown in Equation (2), visualize the trajectory of the embedded time series in an embedding space of a higher dimension. However, given sufficient embedding of the time series, we would also expect that major frequencies/periods of the non-embedded time series are incorporated in the RP, its τ-RR, and eventually in the inter-spike spectrum of the τ-RR. In order to demonstrate this, we apply the inter-spike spectrum to the freely available eccentricity time series of Laskar, J. et al. [33]. This astronomical computation of the orbital motion of the Earth (here, we focus on the eccentricity only) has a clear expectation value of the incorporated frequencies/periods. The three leading eccentricity cycles of 405 kyr period, 95 kyr period and 124 kyr period are well known in palaeoclimate studies [34,35]. Our aim in this section is to show that the inter-spike spectrum of the τ-RR of the embedded eccentricity time series will reflect these cycles in a similar fashion as the Fourier power spectral density of the non-embedded eccentricity time series. We will further show that Fourier-transforming the τ-RR instead of applying the proposed inter-spike spectrum will lead to non-satisfying results, since the spiky τ-RR excites a variety of harmonics in the corresponding Fourier spectrum (cf. Section 1). We use a time series which covers the past ∼67 Mio. years (Myr), with a total length of N=13,421 samples and a sampling period of Δt=5000.

**Figure 8:**
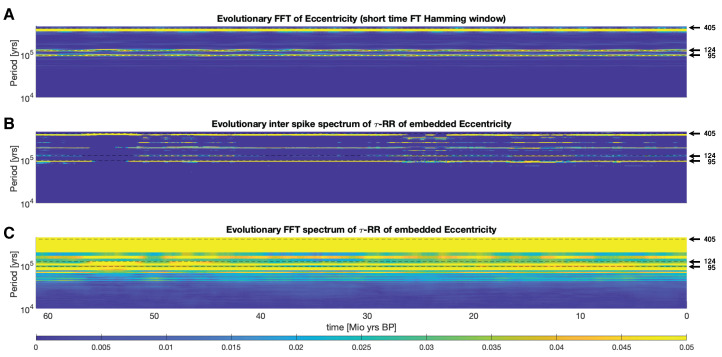
(**A**) Evolutionary Fourier power spectra of eccentricity time series. (**B**) Inter-spike spectrogram of the τ-recurrence rate of the eccentricity time series and (**C**) its Fourier spectrogram. Horizontal black dashed lines highlight the analytically expected orbital periods of 405, 124 and 95 kyrs. For comparability, in all cases the spectra aligned in the columns of the shown plots are normalized to probabilities (sum of unity for each power spectrum). For further computational details, please refer to the main text.

First, we compute an evolutionary short time FT using a windowsize of ws=1000 samplepoints (≡5 Myr) shifted by unity and a Hamming window. The spectrogram reveals the expected periods mentioned, which are highlighted and clearly visible (Figure 8A).

Then, we construct the inter-spike spectrogram of the τ-RR by first determining an appropriate embedding. By using a recent tree-embedding ansatz [36], we minimize the *false nearest neighbor* statistic [37] and use the continuity statistic [38] for potential delays. Eventually, we obtain 8-dimensional embedding with embedding delays τ=[0,18,36,48,62,101,114] (in sampling units) for the entire time series. Similar to the preceding approach we embed the time series with these embedding parameters on windows of size ws=1,200 and a unity shift. (We use a slightly larger window here than in the short FT, because the embedding causes a “loss” of data points and we want to cover similar time spans.) The RPs are computed on these embedded trajectories with a fixed recurrence threshold corresponding to a 10% global recurrence rate [10] and the inter-spike spectra of the corresponding τ-RRs is obtained by using STLS regression (see Section 2) and a regularization threshold corresponding to ρ=0.9 accordance of τ-RRs and re-composed signals. We only use the first 200 data points of the τ-RR (covering a time span of 1 Myr). The spectrogram also highlights the 95, 124 and 405 kyr periods as expected (Figure 8B). Additional power is distributed in the harmonics at 190 and 248 kyr periods. Finally, the standard FT with a Hamming window of the same τ-RRs used to obtain the inter-spike spectrogram in panel B yields a smeared spectrogram which does not reflect the expected periods, but rather suffers from the spike train behavior, i.e., many excited harmonics, of the FT described in Section 1 and Figure 2. The shown results are robust to a change of the embedding parameters and windowsizes. However, a low regularization threshold smears the clear spectrogram in panel B.

## 4. Discussion

We successfully used the idea of transforming the τ-RR for the detection of bifurcations in the Logistic map. By constructing appropriate surrogates of the inter-spike spectra, and, thus, a null model, the number of significant peaks in the inter-spike spectrum correlated well with the positive Lyapunov exponent. This measure was also able to resolve period-doubling bifurcations. However, we have to admit that by using this method, the detection of a bifurcation is only possible when the additional period(s) is not an integer multiple of the former period(s). This behavior is described in the application to the Rössler system, where we explicitly showed the different inter-spike spectra for period-2, period-3, and chaotic dynamics. Further development might potentially incorporate the mutual distance of peaks in the spectrum for a better correlation to the Lyapunov exponent. The inter-spike spectra of power grid frequency data illustrate that our proposed method may serve as a valuable source of information in addition to a standard Fourier analysis. Last but not least, we showed that our approach incorporates frequencies, which are apparent in the Fourier spectrum of the signal, by applying the method to analytically derived eccentricity data, where the dominant frequencies are well known.

**Figure A5:**
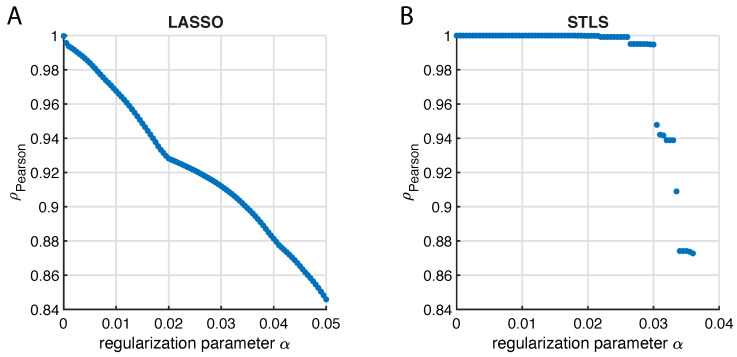
Pearson correlation coefficient $\rho_{\vec{s},\tilde{\vec{s}}}$ between the input signal signal $\vec{s}$ and the re-composed signal $\tilde{\vec{s}}=X^{T}\hat{\beta }$ as a function of the regularization parameter α for (**A**) LASSO and (**B**) sequentially thresholded least squares (STLS) regression (see also Section 2). The input time series is of length N=200 and stems from the τ-RR of Rössler system in regular dynamics.

We discuss some more technical details in the following sections, which will also affect any application of the proposed method. First of all, the number of required basis functions $M=\sum_{i=1}^{\left\lceil N/2\right\rceil +1}i$ for an input signal of length *N* is the crucial bottleneck of this approach, which is why it does not show good scaling behavior. The subsequent sparse regression, therefore, becomes computationally intensive for N>1000. Depending on the memory of the computer used, input signals N>2000 usually do not work. This means that signals often need to be downsampled as a preprocessing step (e.g., see Appendix B). Second, the regularization parameter α for the regression is a crucial free parameter. As described in Section 2, our idea in this paper is to select the α such that the re-composed signal $\tilde{\vec{s}}=X^{T}\hat{\beta }$ matches a given (Pearson) correlation coefficient $\rho_{\vec{s},\tilde{\vec{s}}}$ between the original signal $\vec{s}$ and itself. This ensures that α adjusts itself to the data as well as to the used regression method. We found that this increases the comparability of different spectra, especially when performing a running window approach in order to obtain an evolutionary spectrogram (Figure 8). However, the two different sparse regression algorithms we encountered in this article (LASSO and STLS) yield different results for the same desired $\rho_{\vec{s},\tilde{\vec{s}}}$. Even if the spectra obtained in this way look qualitatively similar, they are not always quantitatively similar. The reason for this is that $\rho_{\vec{s},\tilde{\vec{s}}}$ is not a smooth function of α in case of STLS, due to the hard-thresholding involved [25], which is shown in Figure A5. Third, when adopting our idea of applying the inter-spike spectrum to the τ-recurrence rate of the signals state space trajectory, the embedding process induces additional free parameters. This is not a drawback of the proposed decomposition method, but rather a drawback of applying this technique to the τ-recurrence rate of the system, which was the main motivation for developing the proposed method. As a very last remark, we draw attention to the fact that sparse regression can be transformed into sparse logistic regression when the signal we would like to transform is binary.

## 5. Conclusions

A novel decomposition technique is proposed that yields the *inter-spike spectrum*. The method decomposes any arbitrary signal into basis functions which consist of (lagged) Dirac combs (DC) of a different inter-spike period. The loading for each period is obtained by a regularized regression, which promotes sparsity in its solution. We chose LASSO or a sequentially thresholded least squares regression STLS in this letter. Since there are $M=\sum_{i=1}^{\left\lceil N/2\right\rceil +1}i$ basis functions for a signal of length *N*, the regression can become computationally intensive for N>1000. When plotting the computed loadings as a function of the period (or frequency), the inter-spike spectrum is obtained. A disadvantage is that the transformation is not invertible. An advantage is that there is no leakage effect. Although this novel spectrum is superior to an ordinary FFT-based power spectrum when the signal has a spike-train-like appearance, the authors suggest that this method should be considered as an additional source of information but not as a substitute for ordinary Fourier analysis. Due to the sparse regression underlying the method, there is no unique inverse of the transformation and the regularization parameter plays a crucial role and determines the appearance of the obtained inter-spike spectrum. Moreover, similar to the Nyquist frequency barrier in the Fourier Transform which sets a lower bound for the corresponding wave period, here the maximum considered inter-spike period is bounded by $T_{\mathrm{is}}^{\max}=\left\lceil N/2\right\rceil +1$.

The creation of the proposed method was by the idea of transforming τ-recurrence rate signals (τ-RRs) into their frequency domain. This general idea [15] facilitates a frequency analysis of high dimensional systems, because the RP is a representation of the system’s state space trajectory. The τ-RR of a recurrence plot (RP) usually has a spiky shape, especially for map-like data, and the inter-spike spectrum can reliably reveal the system’s dominant frequencies, which is not possible when Fourier transforming the τ-RR or the underlying signal itself. Since the position of the peaks in the τ-RR are not sensitive to noise, the corresponding inter-spike spectrum also yields robust results in the presence of noise.

There is a broad range of applications for the proposed idea. The inter-spike spectrum itself can serve as a valuable tool for the analysis of any sort of spike-train-like data. On the other hand, the inter-spike spectrum of the τ-RR of a signal can serve as a generalized, nonlinear frequency analysis tool for complex systems. When there is only a subset of state variables available, the state space has to be reconstructed as a pre-processing step. Recent findings [16,36] show that this reconstruction process can be reliably automated and applied to multivariate data as well. This would allow for a “running window” approach, in order to detect transitions. Due to the mentioned computational constraints of our proposed method, a window size w≤1000 would possibly suffice for most data, especially when it is map-like, i.e., not highly sampled.

## Figures and Tables

**Figure A2:**
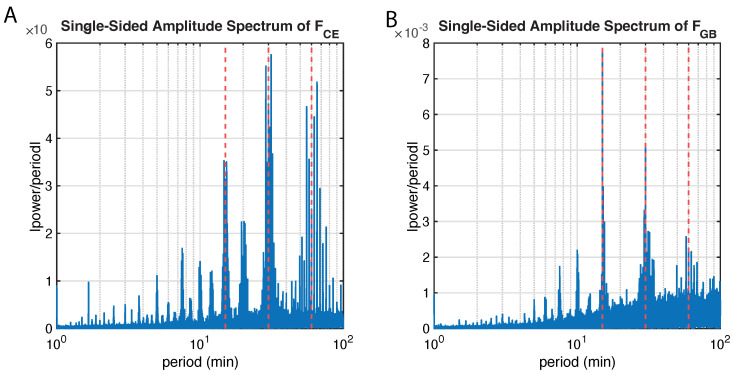
Fourier spectra of the frequency entire time series from (**A**) Central Europe (CE), $N_{\mathrm{CE}}=587,520$ and (**B**) Great Britain (GB), $N_{\mathrm{GB}}=1,581,120$. A subset of the underlying time series is shown in Figure A1.

## Data Availability

The study that we present here is available as a fully reproducible code base (https:doi.org/10.5281/zenodo.7328580) and the method will be available in the Julia language (https:doi.org/10.5281/zenodo.7328513) and as a MATLAB^®^ toolbox (https:doi.org/10.5281/zenodo.7328499) (accessed on 30 August 2022).

## Appendix A. Exemplary Models

### Appendix A.1. Lorenz System

The classical Lorenz-63 system [42] is defined as

(A1) $$\begin{array}{ccc} \dot{x} & = & \sigma (y-x)\\ \dot{y} & = & x(r-z)-y\\ \dot{z} & = & xy-\beta z. \end{array}$$

For producing Figure 1, we set the initial condition to $u_{0}=\left[0.0,10.0,0.0\right]$, used a sampling time of Δt=0.01 and discarded the first 2000 points of the integration as transients. The parameters have been set to σ=10,β=8/3,ρ=28 and we used a time series consisting of 6000 samples.

### Appendix A.2. Rössler System

The Rössler system [43] is defined as

(A2) $$\begin{array}{c} \begin{array}{ccc} \dot{x} & = & -y-z\\ \dot{y} & = & x+ay\\ \dot{z} & = & b+z(x-c). \end{array} \end{array}$$

For producing Figure A3 and Figure 4, the initial condition for producing panels **A** & **B** was set to $u_{0}=\left[0.7,-1,0.4\right]$ with a sampling time of dt=0.05 and in case of panel **C**, $u_{0}=\left[-0.1242,-2.5415,0.2772\right]$ with a sampling time of dt=0.1. The first 5000 samples were discarded as transients and trajectories of length N=5000 were obtained from which we computed the RPs and the corresponding τ-RRs. For the inter-spike spectra, only the first 1000 values of the τ-RRs were considered.

## Appendix B. Power Grid Frequency Time Series

The raw frequency time series have a length of ${\tilde{N}}_{\mathrm{CE}}=58,752,000$ and ${\tilde{N}}_{\mathrm{GB}}=31,622,400$ with sampling times $\Delta t_{\mathrm{CE}}=0.2$s and $\Delta t_{\mathrm{GB}}=1$s, respectively. GB frequency data were measured during 2016, and were obtained from [44]. CE frequency data were from 2017 and were obtained from [45].

We downsampled these time series to a sampling time of $\Delta t_{\mathrm{CE}}=\Delta t_{\mathrm{GB}}=20$s, which led to total time series lengths of $N_{\mathrm{CE}}=587,520$ and $N_{\mathrm{GB}}=1,581,120$ which we used for further analysis.

In this analysis, we divided the time series into non-overlapping blocks of length $N_{\mathrm{block}}=4320$, covering a time span of 24 h. For each time series block we computed Fourier spectra and recurrence plots (RPs), Equation (1), along with their τ-RRs, Equation (2). The RPs were obtained from a uniform 5-dimensional time-delay embedding of each time series with the delay set to the first minimum of the mutual information [37,46] and a fixed recurrence threshold corresponding to an 8% recurrence rate was used in order to ensure comparability [10]. The first 600 data points of the τ-RRs were used to obtain inter-spike spectra with a LASSO regression and a regularization threshold corresponding to ρ=0.95 accordance of τ-RRs and re-composed signals. The spectra shown in all panels of Figure 7 are the averages over all blocks, following Meyer et al. [31].
